# Ecological stoichiometric characteristics of Carbon (C), Nitrogen (N) and Phosphorus (P) in leaf, root, stem, and soil in four wetland plants communities in Shengjin Lake, China

**DOI:** 10.1371/journal.pone.0230089

**Published:** 2020-08-06

**Authors:** Dagne Tafa Dibar, Kun Zhang, Suqiang Yuan, Jinyu Zhang, Zhongze Zhou, Xiaoxin Ye

**Affiliations:** School of Resources and Environmental Engineering, Anhui University, Hefei, China; Assam University, INDIA

## Abstract

Ecological stoichiometric should be incorporated into management and nutrient impacted ecosystems dynamic to understand the status of ecosystems and ecological interaction. The present study focused on ecological stoichiometric characteristics of soil, and leaves, stems, and roots of different macrophytes after the banning of seine fishing in Shengjin Lake. For C, N, and P analysis from leaves, stems, roots, and soil to explore their stoichiometric ratio and deriving environmental forces, four dominant plant communities (*Vallisneria natans*, Zizania *latifolia*, *Trapa natans and Carex schmidti*i) were collected. The concentration of C, N, P and C: N: P ratio in leaves, stems, roots, and soil among the plant communities varied significantly. Along the depth gradient high C: N was measured in *C*.*schmidtii* soil (7.08±1.504) but not vary significantly (*P* >0.05). High C: P result was found in *T*.*natans* (81.14±43.88) and in *V*.*natans* soil (81.40±42.57) respectively with no significant difference (p>0.05). Besides, N: P ratio measured high in *V*. *natans* (13.7±4.05) and showed significant variation (P<0.05). High leaf C: N and N: P ratio was measured in *C*. *schmidtii* and *V*. *natans* respectively. Nevertheless, high leaf C: P ratio was measured in *Z*. *latifolia*. From the three studied organs, leaf C: N and N: P ratio showed high values compared to root and stems. The correlation analysis result showed that at 0-10cm depth soil organic carbon (SOC) correlated negatively with stem total phosphorus (STP), and root total nitrogen (RTN) (*P*<0.05) but positively strongly with leaf total phosphorus (LTP) and leaf total nitrogen (LTN) (*P*<0.01) respectively. Soil total nitrogen (STN) at 0-10cm strongly positively correlated with leaf total phosphorus (LTP) (*P*<0.01) and positively with RN: P and leaf total carbon (LTC) (*P*<0.05). Soil basic properties such as soil moisture content (SMC), bulky density (BD) and pH positively correlated with soil ecological stoichiometric characteristics. Redundancy analysis (RDA) result showed available nitrogen (AN), soil total nitrogen (STN), and available phosphorus (AP) were the potential determinants variables on plants stoichiometric characteristics.

## Introduction

Ecological stoichiometry is an important tool for studying ecological processes and functions [1]. Besides, it uses to explore the dynamic balance of various elements and their interactions [2] and limitation of plant growth [3]. Carbon (C), Nitrogen (N) and phosphorus (P) concentrations are vital element sources in plant and their changes in characteristics limit plant growth [4]. Further, Nitrogen (N) and Phosphorus (P) elements are determinant nutrients for plant growth and aquatic ecosystems functioning [5] and their mass ratio can potentially affect plant-mediated ecological processes [6]. Soil C: N: P ratios can directly reflect soil fertility and indirectly the nutritional status of plants and species composition of plant communities [7]. Carbon, nitrogen, and phosphorus biogeochemical circulations closely related to the soil's ecological structure, processes, and functions in wetland ecosystems [8]. However, it varies due to the difference in vegetation characteristics [9] plant identity associated with growth rate, and nutrients allocation [10], and plant size, taxa, and life forms [11]. Recently stoichiometry of C: N: P has been applied to understand nutrients limitation, community dynamism [12], nutrient use efficiency [13] and the global biogeochemical cycle [14] in both terrestrial and aquatic ecosystems. Moreover, plant C: N: P stoichiometry is strongly influenced by nutrient availability, and can effectively show the changes in C, N, and P cycles [15], control plant functional type, climate and anthropogenic interference [16]. Ecological stoichiometry especially plants leaf C, N, and P plays a vital role in analyzing the composition, structures, and function of the concerned community and ecological systems [11, 17]. Under different environmental conditions, plant physiological processes [18], wetland hydrology, soil pH [19] and salinity [20], and community type [21] can determine these nutrients and in tur n, it can be used for the accumulation and allocation of plant biomass [22]. Because of human interference, climate change, hydrological fluctuation, aquatic and offshore environment wetlands have been greatly affected [9]. These are greatly affecting aquatic vegetations and enhancing ecosystem degradation to the maximum peaks [23]. As a result, C: N and N: P ratio which can be used as effective indicators for the health conditions and growth status of plants [16, 24] and understating the life strategies of plants [25] are important as it reflects causes and consequences of ecological integrity. This mainly provides good information to know different plants adaptation capacity to changing environment and stress [26]. Submerged plant communities have dominated our study site before 2008 as [27] reported in his study. However, due to overfish production and using seine fishing net they have been drastically affected. Thus, the government officially banned using seine fishing since 2008 and those drastically disappeared vegetations began restoring gradually. This ongoing action strategy has been designed to manipulate the physical, chemical or biological features of our study site with the goal of returning natural and historical functions of the degraded wetlands formerly. To our knowledge, there was no study conducted on the ecological stoichiometry of wetland soil in this Lake after the regeneration of those degraded plants and banning of using seine fishing net. With this knowledge gap, this study mainly sought to determine, (1) the distribution patterns and stoichiometric characteristics of C,N, P, and C:N:P in soil, leave, stems, and roots of different macrophytes communities (2) to analyze soil-plant ecological stoichiometric interaction.

## Materials and methods

### Study site description

**Fig 1:**
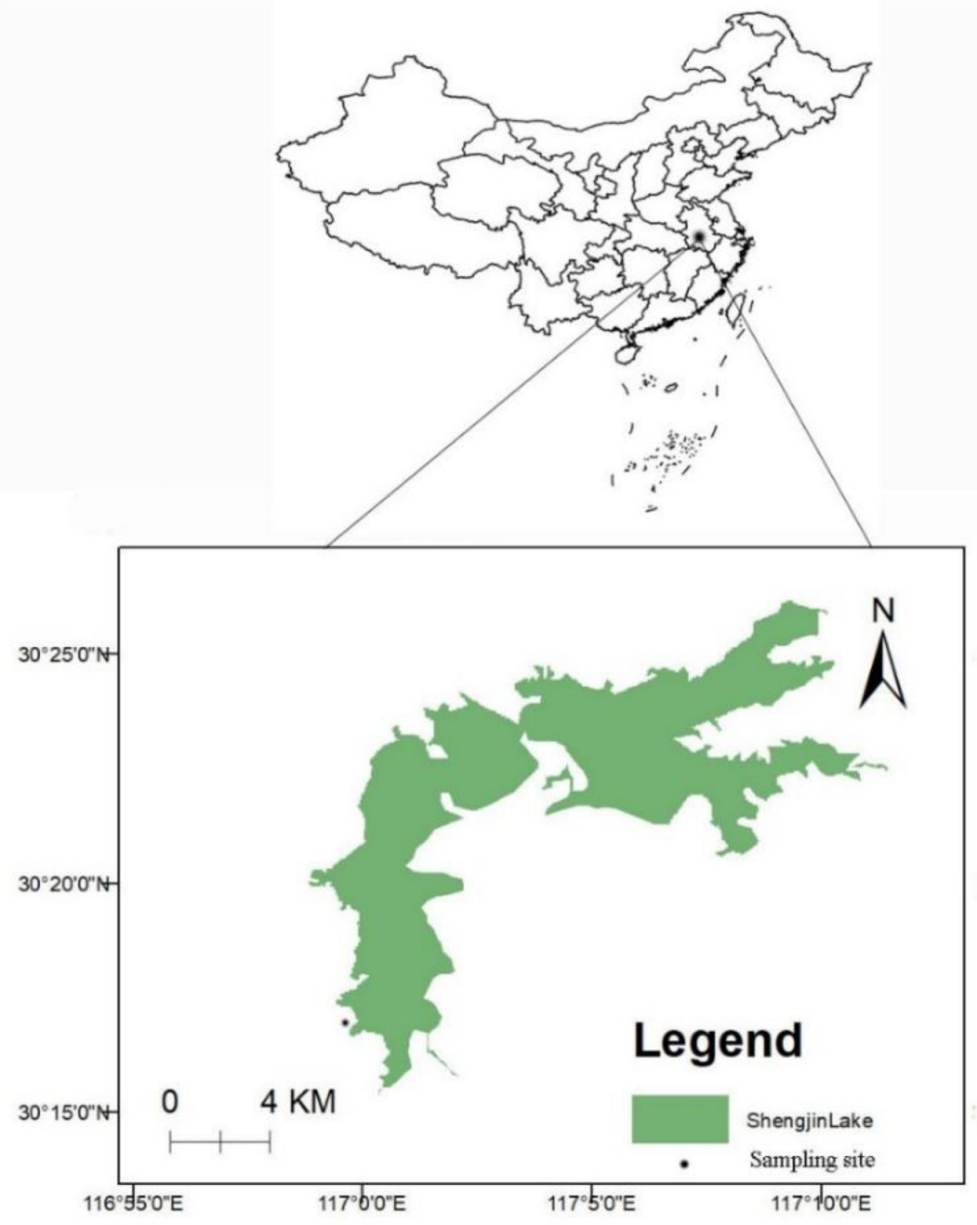
Sampling site geographical location.

Shengjin Lake is located at (30°16′–30°25′N, 116°59′–117°12′E), in the southern bank of the Yangtze River, close to Anqing (Fig 1). It has an area of 133 km^2^ in the wet and 33 km^2^ in the dry seasons respectively. The protected area is centered on Shengjin Lake and the coast extends outward by about 2.5 km^2^. The maximum area of the lake during the flood peak is ~14000 ha (17.0 m (asl) but, the water level normally falls each year to less than 10 m (asl) during the dry seasons and decrease to ~3400 ha. It gets inflow from three small rivers flowing directly into the lake and from the Yangtze River via the Huangpen Sluice. Various terrains with low mountains, hills in the southeast, and plains in the northwest surround the lake.

### Soil and plant sampling

To examine ecological stoichiometry of C, N, and P in soil, the soil samples were collected from four macrophyte plants having various life forms via *Zizania latifolia* Turcz., (emergent), *Vallisneria natans* Lour., (submerged), *Trapa natans* L., (floating-leaved plant) and *Carex schmidtii* Meinsh., (emergent marginal wetland plant) respectively. Those studied macrophyte taxa have been selected based on the areal coverage and, minor aggregated macrophytes groups were excluded in this study. The sampling area was restricted to the area where highly exposed to fishing activity and this action caused the disappearance of those regenerated taxa after the banning of seine fishing. Thus, the sampling was confined to this targeted sampling area to know the effect of management action taken yet in relation to the banning of seine fishing net and regeneration of those disappeared macrophyte taxa targeted in this study. To fix the area of sampling plot 1m×1m framed quadrat was used and the samples were collected using a 5cm diameter soil auger in October 2019 randomly after clearing the selecting sites. Soil sampling profile sectioned into 10cm interval as 0-10cm, 10-20cm, and 20-30cm in depth vertically after [25]. In each depth and sampling plots, three replicate samples were taken and mixed evenly to homogenize and composited. The homogenized soil samples were grouped per specific plant communities, placed in polyethylene plastic bags, and transported to the laboratory for analysis. For plant total carbon (PTC), plant total nitrogen (PTN), and plant total phosphorus (PTP) analysis, we collected root, stem, and leaf. The root samples were uprooted properly and cut off with scissors, washed with hot tap, and distilled water for analysis in the laboratory [28]. The leaves and stems surface were washed by tap, and distilled water in the laboratory to avoid epiphytic and adhering muds to the surface.

Field sampling for this site data collection was permitted by Mr. Zhongze Zhou.

### Laboratory analysis

At room temperature, both soil, plant leaf, stem, and root samples were air dried in open space. From the homogenized soil samples visible stones, rocks, shells, plant debris, and roots were removed by hand. The air dried soil samples were crushed and ground using mortar and pestle and sieved through 0.15mm sieve before analysis. Leaf samples (without petiole and rachis), stem, and root were ground using a ball mill after [29] and sieved through 0.15mm sieve to analyze carbon, total nitrogen, and total phosphorus. All samples were triplicated and averaged procedurally during analysis. Carbon contents in soil, leaf, stem, and root were treated by wet oxidation of organic matter with potassium dichromate (K_2_Cr_2_O_7_) solution and sulfuric acid (H_2_SO_4_), followed by ferrous sulfate (FeSo_4_) as titrant [30]. Analysis of total nitrogen (TN) from soil, leaf, stem, root, and available nitrogen (AN) was carried out using perchloric acid (HClO_4_) by digesting with sulfuric acid (H_2_SO_4_) and measured by UV-2450 spectrophotometer (Shimadzu Scientific Instruments, Japan) [31]. After digesting by sulfuric acid (H_2_SO_4_) and hydrogen peroxide (H_2_O_2_) a TU-1901DS ultraviolet spectrophotometer UV-2300 (Tec comp Com, Shanghai, China) molybdenum antimony colorimetric method was used to quantify soil, leaf, stem, and root total phosphorus (TP) and available phosphorus (AP) [30]. Finally, C, N and P concentrations were presented as g kg-^1^ on a dry mass basis [22] and available (AN) and available phosphorus (AP) in (mg kg-^1^). The stoichiometry of C: N: P molar ratios were computed as mass ratio proportion. Portable pH meter (Sensor, Hach, USA) was used to measure soil pH and Electrical conductivity (EC) after the samples have been made 1:5 soil/water ratio in deionized water in the suspension after mixing the samples for one hour intermittently [32]. Core soil moisture contents (SMC) and bulk density (BD) were determined by the oven-dry method [30].

### Quality assurance

To ascertain our experimental quality at the time of sample digestion and processing, we used the blank sample to manage the background effect of materials used for each analyzed sample. Besides, all used sampling bottles have been soaked in acid solution (HCl) from 30–40 minutes, washed in deionized water and oven dried before use accordingly.

### Data analysis

One way analysis of variance (ANOVA) was applied for the statistical significance test. Mean and the standard deviation was used to describe all variables in the statistical analysis and mean values were reported at 95% confidence interval. Person correlation coefficient analysis was used to test the relationship between total organic carbon (TOC), total nitrogen (TN), total phosphorus (TP), and C: N: P ratios in leaf, stem, root, soil and environmental variables in different plant communities at (p<0.05). The data were tested for normality distribution and homogeneity of variance before analysis has been carried out. SPSS.20 (IBM Corporation, Armonk, New York) software was used for all statistical analyses. RDA was carried out using Canoco 4.5 for Windows (Microcomputer Power, Ithaca, NY) to select the best explanatory variables. The data and Monte Carlo reduced model tests with 499 unrestricted permutations was used to statistically evaluate significance. All columnar figures were done using Graph Pad Prism 5 (https://www:graphpad.com)software) software.

## Results

### Soil C, N and P distribution patterns among different plant communities

**Fig 2:**
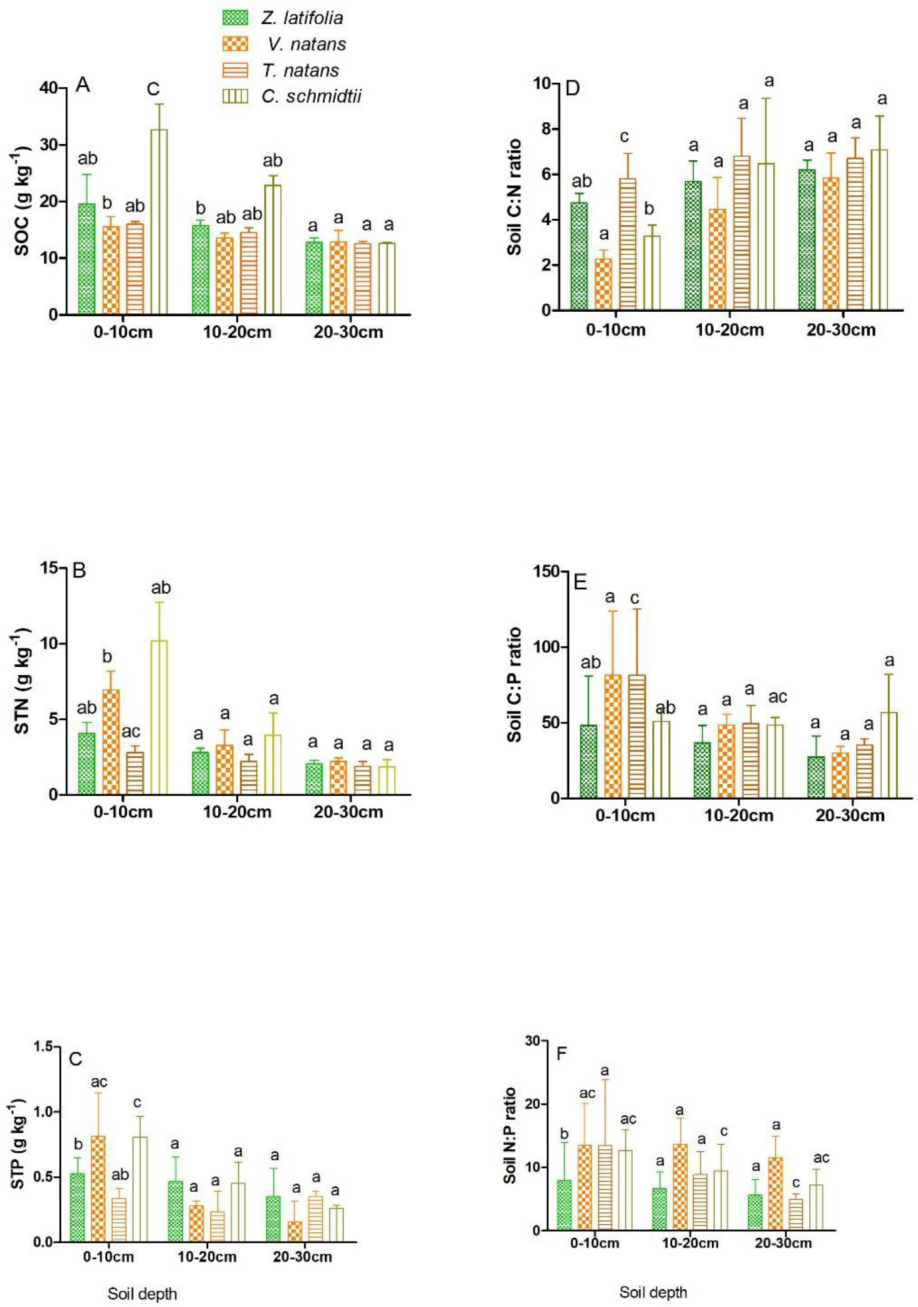
The vertical distribution of soil organic carbon- SOC -(2A), soil total nitrogen STN- (2B), soil total phosphorus STP- (2C), C:N-(D), C:P-(2E), N: P-(2F). Figures represent Mean±SD, different letters show significant difference at p <0.05.

Different vegetation types had significantly affected soil organic carbon (SOC), soil total nitrogen (STN), and soil total phosphorus (STP) distribution and varied significantly (P <0.05) (Fig 2A, 2B and 2C) along the depth gradients. As illustrated in (Fig 2A), soil organic carbon (SOC) in plants communities varied from (32.6±4.59–12.56±0.342 g kg^-1^). Soil organic carbon (SOC) content was ordered among studied macrophyte taxa as *Carex schmidtii* > *Zizania latifolia*> *Vallisneria natans* > *Trapa natans* respectively. In the same manner, the concentration of soil total nitrogen (STN) was ranked from (1.84±0.474–10.18±2.56 g kg^-1^). Spatially, high soil total nitrogen (STN) was measured in *C*. *schmidtii* followed by *V*. *natans* and *Z*. *latifolia* (6.953±1.23g kg^-1^) and (4.0.74±0.734 g kg^-1^) from 0–10cm soil layer respectively (Fig 2B) and showed significant difference (P<0.05). On the other hand, along the depth profiles the soil total phosphorus (STP) values was found within (0.814±0.3302–0.1594±0.1581 g kg^-1^). Among the four macrophyte taxa in this study, high soil total phosphorus (STP) was obtained in *V*. *natans* soil especially at 0–10cm depth and differed significantly (P <0.05), (Fig 2C).

### Soil C: N, C: P, N: P ratios distribution pattern in four plant communities vertically

Ecological stoichiometric distribution of C: N, C: P and N: P ratios in different plant communities were found vary significantly along the vertical distribution at different depths. Of these, C: N ratio was ranked within (2.28±0.392–7.084±1.504) and slightly high in C. schmidtii without any significant difference (P >0.05) (Fig 2D). Furthermore, the ratio of C: P and N: P spatial variation have been illustrated in (Fig 2E and 2F). In average, C: P marked within (27.48±13.78–81.45±43.88) whereas N: P ratios were found from (3.78±2.09–13.7±4.05) respectively. Among the four plant communities high C: P values which indicated more or less similar was measured in *T*.*natans* (81.14±43.88) and in *V*.*natans* soil (81.40±42.57) respectively (Fig 2E) with no significant difference (p>0.05). Likewise, N: P ranged from (3.78±2.09–13.66±4.05) and relatively high value was obtained in *V*.*natans*. Contrarily to C: N, high C: P and N: P ratio result were measured in the surface and middle depth layers than the last depth (20-30cm) vertically.

### Leaf, stem and root ecological stoichiometry pattern in four plant communities

**Fig 3:**
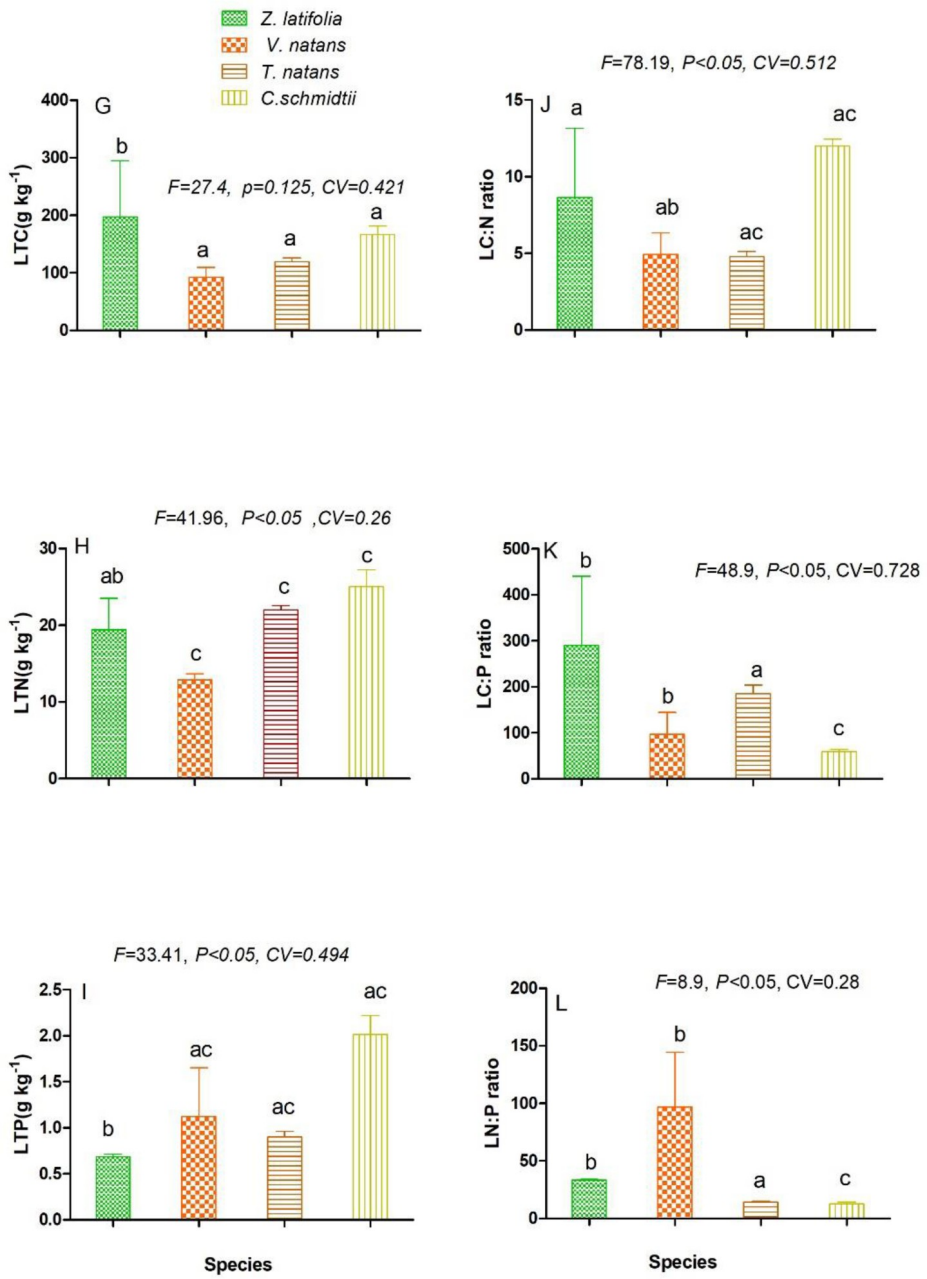
Ecological stoichiometric characteristics of leaf C, N, P and their mass ratio among the four studied macrophytes species. LT-leaf total carbon-(3G), LTN-leaf total nitrogen-(3H), LTP-leaf total phosphorus-(3I), LC: N leaf C: N ratio(3J), LC: P leaf C: P ratio,(3K), LN: P leaf N: P ratio(3L). Figures represent Mean±SD, different letters show significant difference at p <0.05.

**Fig 4:**
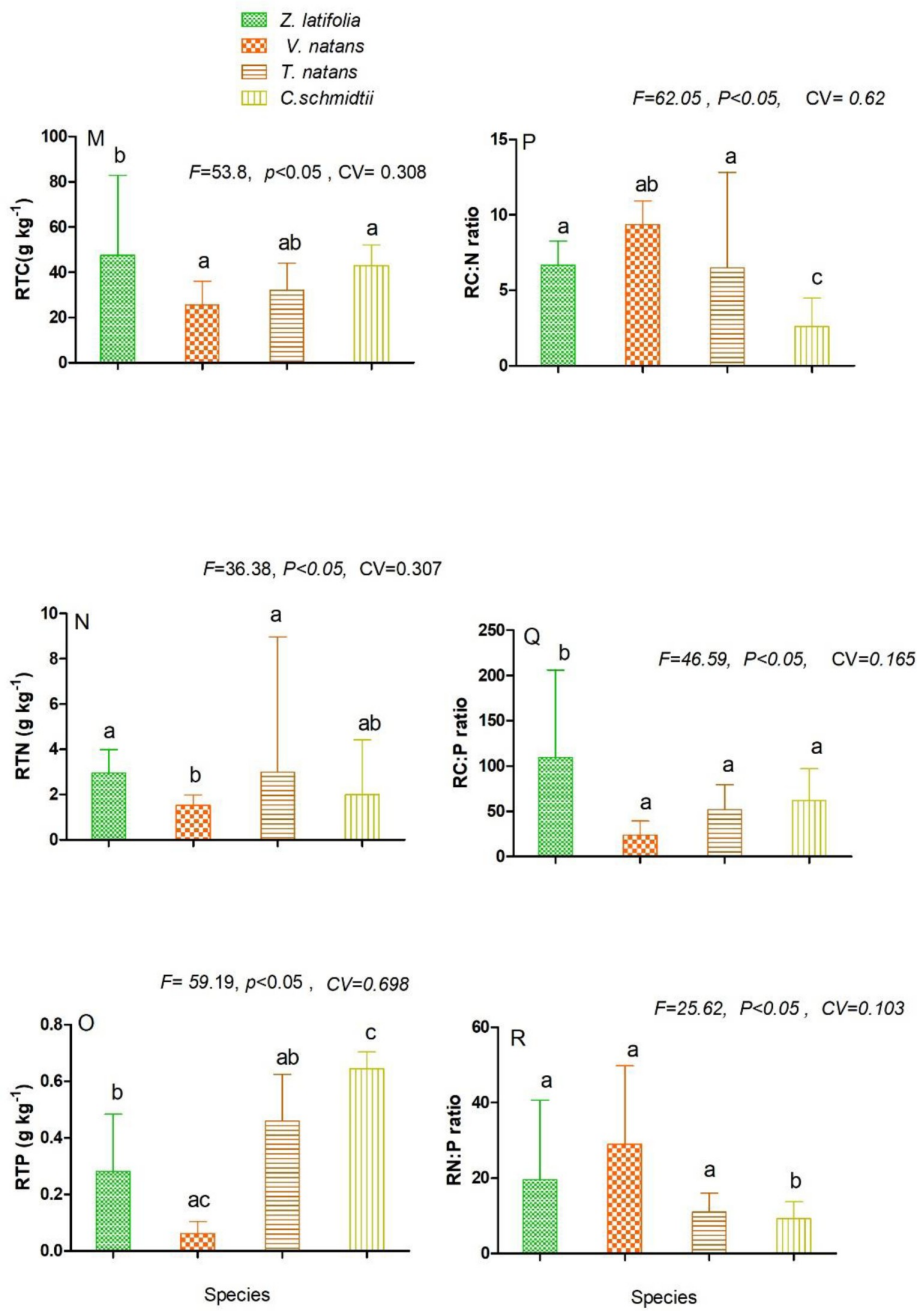
Ecological stoichiometric characteristics of roots C, N, P and their mass ratio among the four studied macrophytes species. RTC-root total carbon-(4M), RTN-root total nitrogen-(4N), RTP-root total phosphorus-(4O), RC: N- root carbon to nitrogen ratio-(4P), RC: P- root carbon to phosphorus ratio-(4Q), RN: P- root nitrogen to phosphorus ratio—(4R). Figures represent Mean±SD, different letters show significant difference at p <0.05.

**Fig 5:**
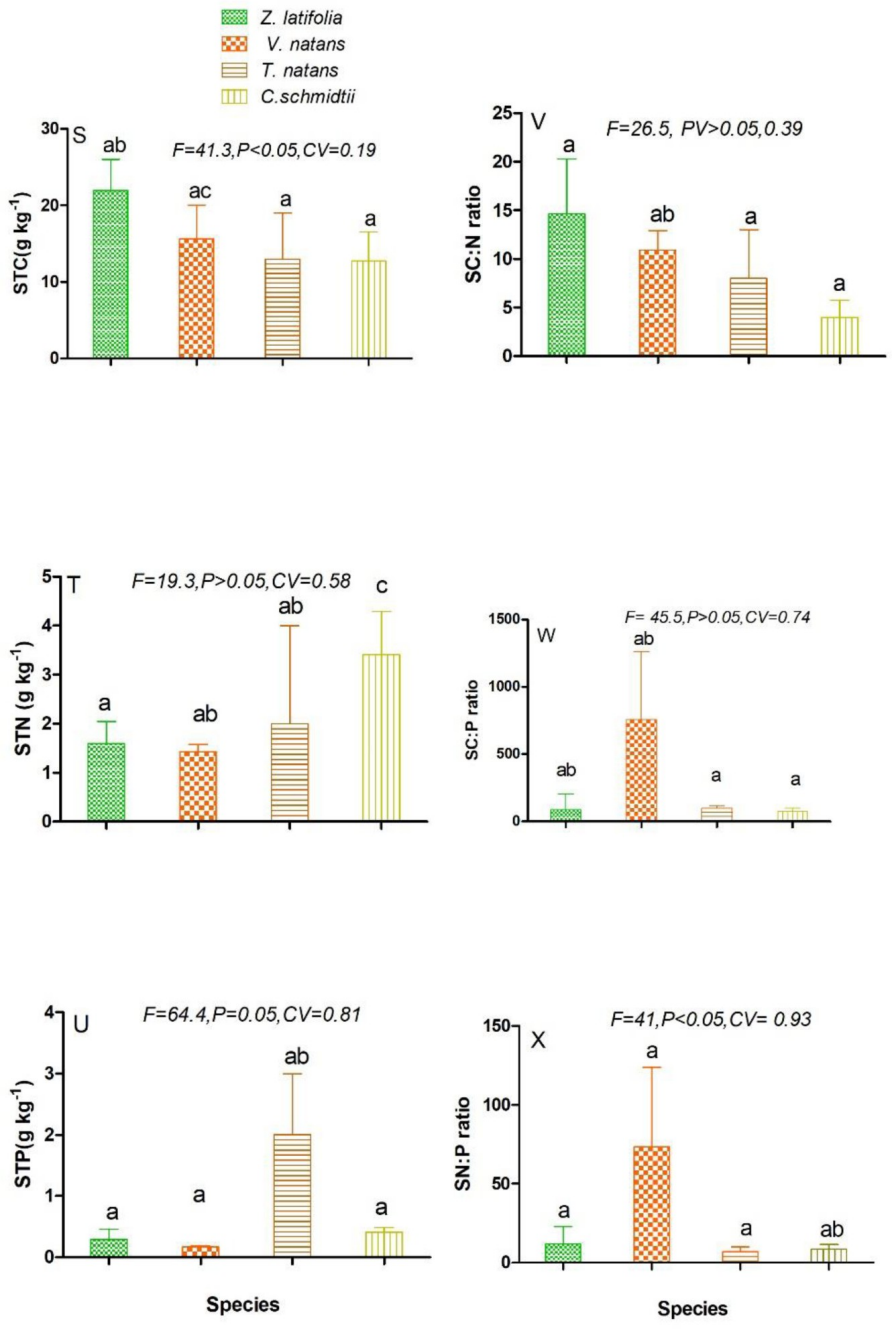
Ecological stoichiometric characteristics of stem C, N, P and their mass ratio among the four studied macrophytes species. STC-stem total carbon-(5S), STN-stem total nitrogen-(5T), STP- stem total phosphorus-(5U), SC: N- stem carbon to nitrogen ratio-(5V), SC: P- stem carbon to phosphorus ratio-(5W), SN: P-stem nitrogen to phosphorus ratio-(5X). Figures represent Mean±SD, different letters show significant difference at p <0.05.

The concentration of total carbon (TC), total nitrogen (TN), and phosphorus (TP) among the tested organs varied among the studied macrophyte taxa (Fig 3G,4M,5S,3H,4N,5T,3I,4O and 5U) respectively. Accordingly, the mean leaf total carbon (LTC), leaf total nitrogen (LTN) was found (92±17.45–197±98 g kg-1, 12.88±0.792–25±2.22 g kg-1) (Fig 3G and 3H) respectively. While leaf total phosphorus (LTP) result was reported within the range of (2.01±0.205–0.6841±0.0263 g kg-1) (Fig 3I). Among the four groups of macrophyte taxa, the highest leaf total carbon (LTC) concentration was measured in *Z*. *latifolia* but showed no significant difference (F = 27.4, CV = 0.421, P >0.05). However, leaf total nitrogen and phosphorus were showed high in *C*. *schmidtii* and varied significant (F = 41.96, CV = 0.26; P <0.05; F = 33.41, CV = 0.494, P <0.05) respectively. On the other hand, the mean of C: N: P ratios in leaf was marked from (4.92±1.42–9.28±0.827, 82.7±4.606–289±150.59), and (21.4±3.206–96.9±47.57) respectively. Of these, high C: N and N: P ratios were obtained in *C*. *schmidtii* and *V*. *natans*, but, only C: N showed significant difference (P <0.05) respectively. Nevertheless, the C: P in *Z*. *latifolia* with significant difference values (F = 48.9, CV, 0.728, P <0.05). With the same approach, the measured root nutrient content varied significantly among the groups. Root total carbon (RTC), total nitrogen, (RTN) concentration was marked within (11.16±2.08–18.63±5.62 g kg-1, 1.52±0.36–5.974±1.604 g kg-1) range and varied significantly (F = 53.8, CV = 0.308, P<0.05; F = 36.38 CV = 0.307, P<0.05) respectively (Fig 4M and 4N). In addition, root total phosphorus (RTP) ranged within (0.0615± 0.0412–0.644±0.042 g kg-1) (Fig 4O) and showed significant difference (F = 59.19, CV = 0.698, P<0.05). Likewise, root C: P: N, ratios were displayed in (Fig 4P, 4Q and 4R) respectively. Similar to leaf and root nutrients, the concentration of nutrients analysed in stem also noted variable. For instance, stem total nitrogen (STN) concertation measured high in C. schmidtii stem (Fig 5T) with no significant difference (F = 19.3, p>0.05, CV = 0.58) contrarily to the remaining groups. Whereas, stem total phosphorus concentration (STP) was found high in T. natans with slight significant difference (F = 64.4, CV, 0.81, P = 0.05) (Fig 5U).

### The relationship between soil properties and soil ecological stoichiometry in four plant communities

(Tables 1 and 4) showed that there is a considerable significant difference and relationship between soil ecological stoichiometric characteristics and environmental variables respectively. pH values were reported within (5.433±1.10–7.486±0.615) and showed significant difference (P <0.05). Furthermore, pH correlated strongly positively soil organic carbon (SOC) and soil total nitrogen (STN) (P<0.01), but negatively with soil total phosphorus (STP), S C: N and SN: P ratio (P<0.05) (Table 2) respectively. On the other side bulk density (BD) and soil moisture content (SMC) ranged with (0.904±0.256–1.48±0.213) and (30.01±14.82–57.38±34.29) respectively (Table 3). Moreover, pH, bulk density (BD), and soil moisture content (SMC) were correlated positively with soil organic carbon (SOC), and soil total nitrogen (STN) (P <0.05) respectively (Table 4). However, pH negatively correlated with soil total phosphorus (STP), C: N and N: P ratios (P <0.05). Bulk density (BD) and soil moisture content (SMC) showed negative relationship with C: N and N: P ratios (P<0.05) respectively (Table 4). On the other hand, electrical conductivity (EC) related negatively with soil organic carbon (SOC), soil total nitrogen (STN), and soil total phosphorus (STP) (P <0.05) respectively. Available nitrogen (AN) ranged between (0.351±0.2495–0.602±0.222 mg kg-L), whereas available phosphorus ranged between (24.6±8.63–31.04±7.35 mg kg-L) respectively.

**Table 1:** Summary of Redundancy Analysis (RDA) soil ecological stoichiometry and nutrient concentration in various organs.

	1	2	3	4	Total variance
Eigenvalues	0.317	0.023	0.018	0.002	1.000
Cumulative percentage variance of species data	31.7	32.1	32.1	32.1	
Species-environment correlations	0.571	0.411	0.418	0.165	
species-environment relation	88.9	85.2	92.6	97.3	
Sum of all eigenvalues					1.000
Sum of all canonical eigenvalues					0.321

**Table 2:** Pearson correlation analysis (2- tailed) result between soil ecological stoichiometry and plant nutrients in root, stem and leaf along the depth gradients.

	Soil C			Soil N			Soil P		
	0–10 cm	10–20 cm	20–30 cm	0–10 cm	10–20 cm	20–30 cm	0–10 cm	10–20 cm	20–30 cm
Root C	-0.982*	-0.773*	0.226	0.181	0.999*	0.876*	-0.834*	0.346	-0.252
Root N	-0.998*	0.343	-0.999*	-0.952	0.885	0.238	-0.998*	0.772	-0.431
Root P	0.788	0.096	0.710**	0.984*	0.738*	-0.171	-0.268	0.566	0.694
Root C: N	0.691	0.926	0.625	0.603	0.851**	0.912**	0.895**	0.277	0.998**
Root C: P	0.114	0.133	0.061	-0.893*	-0.819**	0.805	0.944**	0.819**	-0.263
Root N: P	-0.999*	-0.565*	-0.733*	0.965*	0.571**	0.784*	0.964**	0.999*	0.423*
Leaf C	-0.883*	-0.856*	0.080	0.805*	0.738*	0.632*	-0.392	-0.783*	-0.850*
Leaf N	0.922**	0.457	0.962**	0.146	0.939	-0.119	0.890**	0.257**	0.487
Leaf P	0.827**	0.999**	0.828**	0.815**	0.849**	0.784**	0.237	0.673*	-0.366
Leaf C: N	0.793	0.944**	0.492	-0.893*	0.177	0.219	0.368	-0.391	-0.282*
Leaf C: P	0.794*	0.159	0.224	0.115	0.191	-0.962**	0.253	0.368	0.106*
Leaf N: P	-0.936*	-0.757*	0.138	0.083	-0.053*	0.259	0.673*	0.287	0.132*
Stem C	0.36	0.07	0.921	0.083	-0.421*	0.290	0.726*	0.285	0.233*
Stem N	0.168	-0.80**	-0.331*	0.249	0.17	0.223	-0.377*	0.044	-0.648*
Stem P	-0.599*	-0.446*	0.305	0.264	0.191	0.771**	-0.703*	-0.331*	0.054
Stem C:N	0.825	0.408	0.296	0.128*	0.47	0.026	0.101	0.210	0.149
Stem C:P	-0.882	0.799	0.666	0.456	0.327	0.703*	-0.622*	0.218	-0.982**
Stem N:P	-0.582*	-0.804*	0.739	0.249	0.553	0.225	0.015	0.327	0.064*

Correlation with * P <0.05, ** <0.01.

**Table 3:** Basic soil characteristics (Mean±SD) in different plant communities in Shengjin Lake wetland.

Plant communities	EC (μS/cm)	pH	BD (g·cm^-3^)	SMC (%)	AP(mg kg^-1^)	AN(mg kg^-1^)
*caduciflora*	51.05±33.76a	5.93±0.693c	1.23±0.219ac	36.19±9.88a	0.181±0.1031b	28.24±9.26b
*V*. *natans*	64.92±33.96a	6.72±0.544c	1.4±0.254a	57.38±34.29ac	0.602±0.222a	31.04±7.35b
*T*. *quadrispinosa*	48.80±44.7a	7.486±0.615a	0.904±0.256ac	43.81±14.98ab	0.449±0.2648ac	24.6±8.63a
*schmidtii*	28.29±21.24b	5.433±1.10c	1.48±0.213c	30.01±14.82ac	0.351±0.2495ac	27.34±9.75ac
Average	48.02±15.05	6.21±0.639	1.24±0.252	43.19±17.46	0.4112±0.1567	27.80±2.653
*P*	<0.05	<0.05	<0.05	<0.05	<0.05	<0.05
CV	0.356	0.539	0.356	0.742	0.389	0.1198
*F*	28.9	61.12	73.58	56.89	29.78	89.27

Mean with different letter denote significance difference at P <0.05 (2- tailed). EC, electrical conductivity, pH, pH values, BD, bulk density, SMC, soil moisture contents AP, available phosphorus, AN, available nitrogen.

**Table 4:** Pearson correlation analysis (2-tailed) result between soil ecological stoichiometric characteristics and environmental variables.

	pH	SMC (%)	EC(μs cm^-1^)	BD(g cm^-3^)	C(g kg^-1^)	N (g kg^1^)	P (g kg^-1^)	C:N	C:P	N:P
pH	1									
SMC%)	0.798*	1								
EC(μ**s** cm^-1^**)**	0.877*	0.798*	1							
BD (g cm^-3^)	0.812**	0.734*	0.739	1						
C	0.848**	0.794**	-0.719*	0.926**	1					
N	0.939**	0.843**	-0.846*	0.734**		1				
P	-0.853*	0.703*	-0.850*	0.676*			1			
C: N	-0.689*	-0.883*	-0.122	0.775*				1		
C: P	0.178	0.993*	0.214	0.847					1	
N: P	-0.815*	0.172	0.302	-0.802*						1

Correlation with * P <0.05, ** <0.01.

### Soil ecological stoichiometric relationship with plant leaf, stem, and root

**Fig 6:**
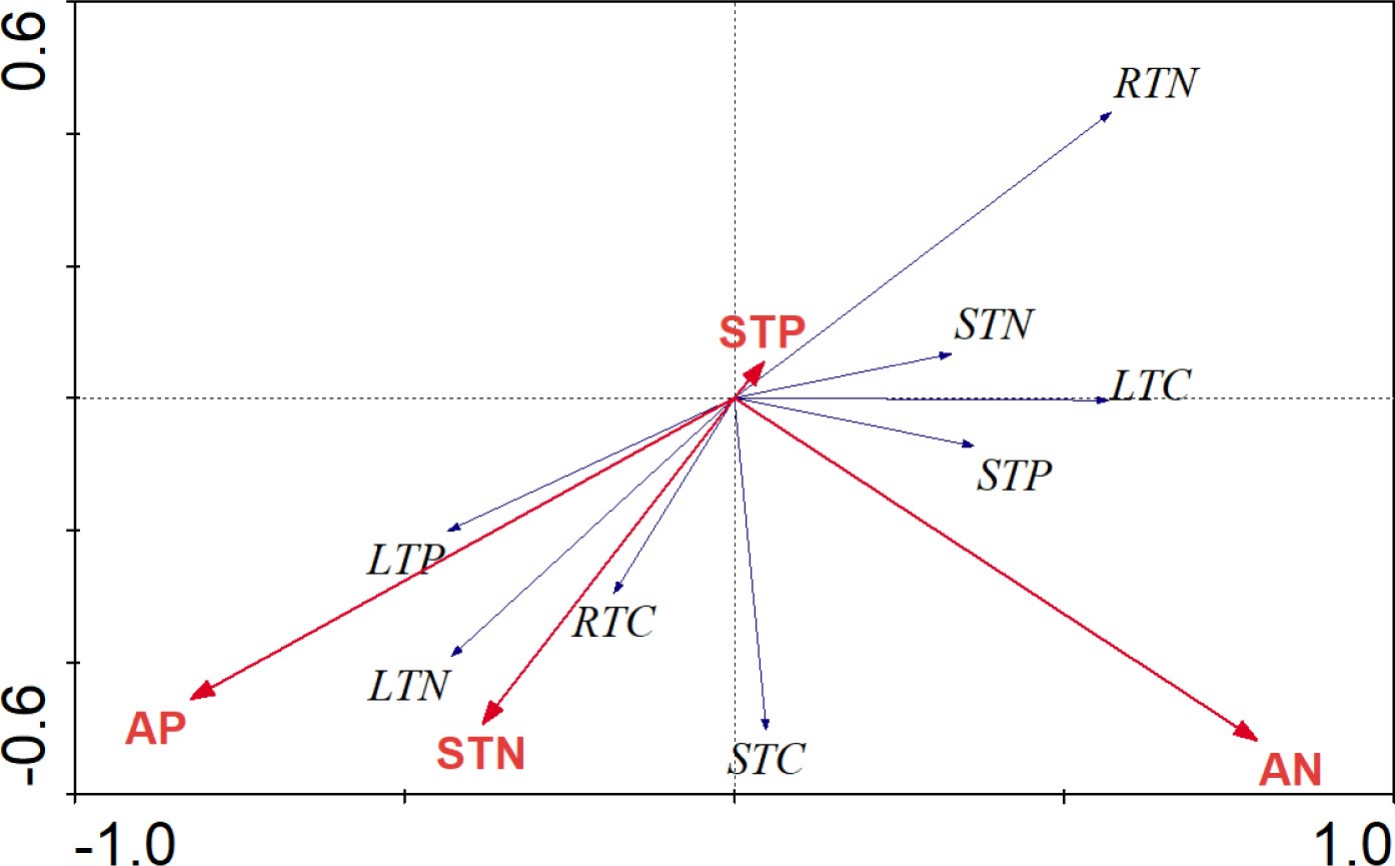
Redundancy Analysis (RDA) result that showed the relationship between soil ecological stoichiometric characteristics and nutrients in various plants organs. The red arrow shows soil ecological stoichiometric characteristics and the blue arrow shows the nutrients concentration in various organs. In the Box plot, LTC, leaf total carbon, LTN, leaf total nitrogen, LTP, leaf total phosphorus, RTC, root total carbon, RTN, root total nitrogen, RTP, root total phosphorus, STC, stem total carbon, STN, stem total nitrogen, STP, stem total phosphorus, EC, electrical conductivity, pH, pH values, SMC, soil moisture contents, BD, bulk density, AN, available nitrogen, AP, available phosphorus.

As reported in (Table 2), soil organic carbon (SOC), soil total nitrogen (STN), and soil total phosphorus (STP) were revealed negative and positive relationship along the depth gradient with leaf, stem, and root nutrients. Of these, at 0-10cm depth range soil organic carbon (SOC), soil total nitrogen (STN), and soil total phosphorus (STP) related positively with leaf total nitrogen, leaf and root total phosphorus (P<0.05), whereas, negatively with root total carbon, root total nitrogen and stem total phosphorus (P<0.05) respectively. However, in the middle depth (10-20cm) soil organic carbon (SOC), soil total nitrogen (STN) and soil total phosphorus (STP) related negatively with root and leaf total carbon, stem total nitrogen, stem total carbon, and stem total phosphorus respectively(P<0.05). Nevertheless, positively correlated with leaf and root total phosphorus, root and leaf carbon, and leaf total nitrogen (P<0.05) respectively. In the same manner, at the last depth profile (20-30cm) soil layer soil organic carbon (SOC) and soil total phosphorus (STP), correlated negatively with root, and stem total nitrogen, and leaf total carbon (P<0.05) respectively. Besides, soil organic carbon (SOC) and soil total nitrogen (STN) associated positively with root, stem, and leaf total phosphorus, leaf total nitrogen, leaf, and root total carbon (P<0.05) respectively. The RDA analysis result indicated soil total nitrogen (STN) and available phosphorus (AP) and available nitrogen (AN) were the most influential environmental variables on root, stem, and leaf nutrients concentration (Fig 6).

## Discussion

### SOC, TN, TP, and C: N: P distribution patterns among different plants communities

The current study indicates that the stoichiometric characteristics in soil and the plant community has a significant effect on the C: N: P stoichiometry characteristics among the four studied wetland macrophytes plants. Along the vertical gradients, soil organic carbon (SOC), soil total nitrogen (STN), and soil total phosphorus (STP) decreased vertically and showed significant difference (P<0.05) (Fig 2A, 2B and 2C) respectively. This result is in agreement with the previous [3, 33] studies. Soil total phosphorus (STP) found high on the surface than the middle and the last depth in this study. [34] reported similar result that may indicate P uptake from deep soil to the surface to meet the nutrient requirements may increase on the surface. Soil nutrients have a strong effect on plant growth and distribution as well as being the primary source determining the concentration of nutrients in plants [30, 35]. In this study, the highest nutrient concentration of soil organic carbon(SOC), soil total nitrogen (STN), and soil total phosphorus (STP) were observed in *C*.*schmidtii* and *V*.*natans* respectively (Fig 2A, 2B and 2C) than the rest two species. This may be related with soil moisture contents as the previous study by [33] report indicated. Besides, in our study result, soil moisture (SMC) showed positive linear correlation between, soil organic carbon (SOC), soil total nitrogen (STN), and C: P ratio (Table 4), which can confirm this conclusion and coincide with the above two cited study results. Moreover, for high soil total phosphorus (STP) measurement in *V*.*natans* than the rest, community relatively may be related with the returning back of above ground residue to the soil and the large number of root decay in wetland ecosystems as indicated by [36]. These factors may contribute their part for high values of total phosphorus (TP) in this species comparatively. High C: P and N: P ratio were found high in *C*. *schmidtii* (81.14±43.88) and *V*. *natans* (13.7±4.05) respectively and varied significantly (P <0.05) (Fig 2E and 2F). Perhaps this may be in relation with their restoring duration and the plant type or particular species are the main factors in affecting C: P ratio [17, 37]. The data presented in this study indicates that, soil stoichiometry at different layers have significantly related with plant stoichiometry (leaf, stem, and root). Soil total nitrogen (STN) showed significantly positive relationship with leaf total phosphorus (LTP) (P<0.001), (Table 2). This result is consistent with [35, 38] study result that implies soil phosphorus concertation has direct effect on the photosynthetically active organ to determine not only phosphorus concentration but also nitrogen level. Root (C: N: P) correlated positively with stem total phosphorus (STP) in our study result. Mainly as the previous studies showed this direct relationship might be linked with the genetic and physiological characteristics of the plant that primarily determine elemental concertation and ratio in their tissues [39, 40]. On the other side, stem (C: P: N) ratio negatively correlated with soil total phosphorus (STP). This may represent tight coupling coordination between soil nutrient and plant stoichiometric characteristics widely. Besides, the growth rhizomes of the plant themselves and the structure characteristics determine plant tissue nutrients [41].

### Soil C, N, P, and C: N: P variation among plant organs and functional unit in different plant communities

The carbon (C), nitrogen (N), phosphorus (P), and C: N: P ratio variation among different organs in different plant communities are affected by both metabolic demand and the functional differentiation, and organizational structure of plant organs [16]. Leaf stoichiometry carbon (C), nitrogen (N), and phosphorus (P) play a vital role in analysing composition, structure, and functions of a concerned community and ecological systems [17]. Leaf total carbon (LTC) was found high in *Z*. *latifolia* in this study. This is in agreement with [41] that the average of C proportion in emergent plant accounts (45%) and in terrestrial plants (50%) [42]. Total nitrogen (TN), and total phosphorus (TP) in different organs were ordered as leaf> root > stem respectively. This may be due to the difference in structure and physiology in different communities. Besides, high leaf total nitrogen (LTN), however, low N: P ratio was measured in *C*. *schmidtii* (Fig 3H and 3L) among the four studied macrophyte plant species. This is supported by [9, 43] report that indicate high N and low N: P ratio concentration, especially in the photosynthetic active organ that species with high growth rates are the best adapted for the environment. This may ascertain the main reason behind the dominance of this marginal plant community especially the beach of the lake, which covers more than 85% (visual estimation) since 2000 on ward. Thus, a high capacity to retain nutrients in biomass and high nutrient use efficiency can thus be a good trait for plants that grow in wetland areas [44, 45] to adapt and increase their size. On the other side, low leaf total carbon (LTC) was measured in *V*.*natans* (Fig 3G). The possible justification for this result is low leaf carbon is due to less lignin and cellulose content in aquatic plants [20] because water buoyancy can provide support for aquatic macrophytes, especially for submerged plants. Next to the marginal wetland plant (*C*. *schmidtii*) which was dominant in our study site, high leaf total nitrogen (LTN) was measured in *T*.*natans* (Fig 3H) compared to the rest remaining species. This is consistent with the previous study by [46] and mainly explained as freely floating macrophytes plant can absorb more N or P from water and sediment through their adventitious root produced from their leaves or stems [47]. There is also significant variation among those studied species with regard to phosphorus (P) and nitrogen (N) concentration in both stem and root parts and as well C:N:P ratios. For instance, high phosphorus (P) and N: P ratio was measured in *V*.*natans* root whereas least in *C*. *schmidtii* (Fig 4O and 4R) respectively. In general, this may show that submersed macrophytes plants can uptake nutrients both from the water column by their leaves or stems and from sediments by their roots or rhizoids [22, 48] these can contribute these two nutrients become high relatively in this species. In ecological studies, leaf N: P ratio was considered as an important index to identify limiting nutrient elements [25, 45]. To grow and function properly plant requires at least 30 elements and any decrease of an element relative their proportion can make limiting their growth. For instance, [25] found if N: P <14 plant growth is limited by nitrogen (N), whereas if N: P >16 plant growth is limited by phosphorus (P) elements in wetland ecosystems. Accordingly, in the present study, the leaf N: P ratio found > 16. This revealed that phosphorus (P) nutrient element was found as a limiting nutrient element than nitrogen (N) in our study.

### The relationship between soil ecological stoichiometry and environmental variables

Basic biogenic elements in soil ecosystems such as C, N, and P were closely related with soil physicochemical properties [11, 24]. In this same way, in this study, soil pH values ranged between (5.433±1.10–7.486±0.615), (Table 3) and positively correlated with soil organic carbon (SOC) and soil total phosphorus (STN) (P<0.01), (Table 4). This may indicate more or less the range of wetland pH values scale that range around 6.5–7.5 with few exceptions in general [49]. In addition, this is in agreement with the previous results reported by [50]. However, pH values showed inverse relationship with soil total phosphorus (STP), C: N and C: P ratios (P<0.05), (Table 4). This is inconsistence with [50] study report which contradict the present study. This may indicate that high pH values in the soil can restrain total nitrogen decomposition [31, 51]. Electrical conductivity (EC) ranged (28.29±21.24–64.92±33.96) (Table 3) and correlated negatively with soil organic carbon (SOC), soil total nitrogen (STN), and soil total phosphorus (STP) (P<0.05) respectively (Table 4). This is coincide with [51] but inconsistent with [52] pervious study report. Soil moisture content (SMC) correlated positively with soil organic carbon (SOC), soil total nitrogen (STN), soil total phosphorus (STP) and C: P ratio (P<0.05) (Table 4) respectively. This is in agreement with [34, 50, 53] studies. Most previous studies indicated basic properties for instance pH, soil moisture content (SMC), bulk density (BD) can regulate the dynamic change of C: N and C: P ratios [54]. Soil moisture content (SMC) positively was correlated with C: P (P <0.05) in the present study. This may indicate high soil moisture content (SMC) can increase organic phosphorus and raise C: P ratio in wetland ecosystems [11]. However, negatively correlated with C: N ratio. This coincide with [7, 50] however, it contradicts [20] previous study result. Bulk density (BD) correlated with soil organic carbon (SOC) and soil total nitrogen (STN) and soil total phosphorus positively (P <0.05) but negatively with C: N ratio (P <0.05) respectively. This result is inconsistent with [50].

## Conclusion and implications

To ascertain healthy and sustainable ecosystems functions, vegetation restoration can play significant roles in the distribution and accumulation of soil and plant stoichiometric characteristics. Thus, in the present study, there is considerable variation in element ratios among the studied macrophyte taxa. The carbon (C), nitrogen (N), phosphorus (P) and C: N: P ratio varied among different organs in four plant communities have been investigated after the lake wetland vegetations began restoring overtime. This is in part caused by the difference in stoichiometric relation between organs that function differently, element availability, and the potential variation in excess uptake difference. Moreover, have also resulted from the functional differentiation, life forms, and organizational structure of plant organs and individual differences among species. High leaf total nitrogen (LTN) and low N: P ratio was measured in *C*. *schmidtii*. This is related to the active organs that provide the best to adapt to the environment. This may ascertain the main reason behind the dominance of this marginal plant community especially at the beach of the lake. High P and N: P ratio was measured in *V*.*natans* root due to their ability to uptake nutrients both from the water column by their leaves or stems and from sediments by their roots or rhizoids. Soil total nitrogen (STN), available nitrogen (AN), and available phosphorus (AP) were found the potential variables that affect the nutrient contents in various organs from the RDA analysis result.
